# Labeling Postsynaptic Densities for Super-Resolution Microscopy With Minimal Signal-Loss and Offset

**DOI:** 10.21769/BioProtoc.5499

**Published:** 2025-11-05

**Authors:** Sheng-Yang Ho, Christiane Huhn, Adam Skeens, Martin Hruska, Hans M. Maric, Johannes W. Hell

**Affiliations:** 1 Department of Pharmacology, University of California, Davis, CA, USA; 2 Rudolf Virchow Center for Integrative and Translational Bioimaging, Julius-Maximilians-Universität (JMU) Würzburg, Würzburg, Germany; 3 Department of Neuroscience, Rockefeller Neuroscience Institute, West Virginia University, Morgantown, WV, USA

**Keywords:** Synaptic markers, Postsynaptic density, PSD-95, Gephyrin, Synthetic peptide probes, Super-resolution probes, STED microscopy probes

## Abstract

Accurate labeling of excitatory postsynaptic sites remains a major challenge for high-resolution imaging due to the dense and sterically restricted environment of the postsynaptic density (PSD). Here, we present a protocol utilizing Sylites, 3 kDa synthetic peptide probes that bind with nanomolar affinity to key postsynaptic markers, PSD-95 and Gephyrin. eSylites (excitatory Sylites) specifically target the PDZ1 and PDZ2 domains of PSD-95, enabling precise and efficient labeling of excitatory postsynaptic density (ePSD). In contrast, iSylites (inhibitory Sylites) bind to the dimerizing E-domain of the Gephyrin C-terminus, allowing selective visualization of inhibitory postsynaptic density (iPSD). Their small size reduces linkage error and enhances accessibility compared to conventional antibodies, enabling clear separation of PSD-95 nanodomains in super-resolution microscopy. The protocol is compatible with co-labeling using standard antibodies and integrates seamlessly into multichannel immunocytochemistry workflows for primary neurons and brain tissue. This method enables robust, reproducible labeling of excitatory synapses with enhanced spatial resolution and can be readily adapted for expansion microscopy or live-cell applications.

Key features

• Protocol for using synthetic bidendate peptide probes: eSylites [1] for PSD-95 at excitatory synapses and iSylites [2] for Gephyrin at inhibitory synapses.

• Compatible with standard antibody staining for multiplexed imaging of primary neurons and tissue sections.

• Reduces linkage error, improving spatial resolution and nanodomain visualization in super-resolution microscopy.

## Graphical overview


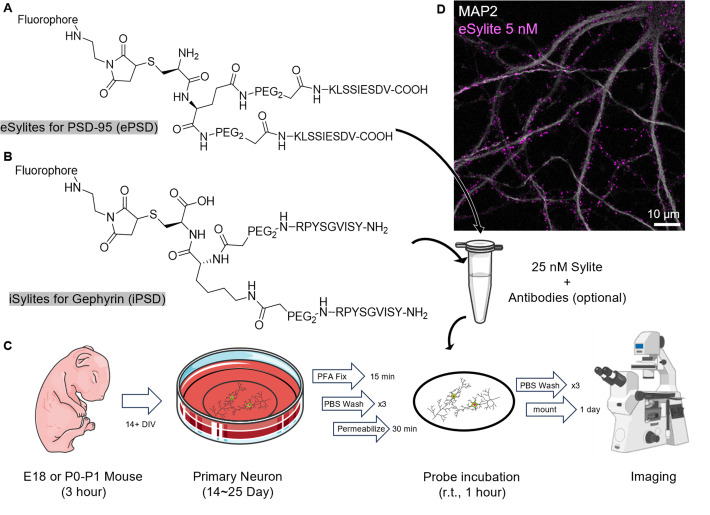


**Graphical overview of the staining procedure.** (A) Structure of eSylite targeting PSD-95 at the excitatory postsynaptic density (ePSD) [1]. (B) Structure of iSylite targeting Gephyrin at the inhibitory postsynaptic density (iPSD) (C) Schematic of Sylites application in fixed primary neuron imaging; more images can be found in Figure 2. Image adapted from Servier Medical Art (https://smart.servier.com/), licensed under CC BY 4.0 (https://creativecommons.org/licenses/by/4.0/). (D) Confocal image of 5 nM sulfoCy5-eSylite on 22 days in vitro (DIV) dissociated hippocampal neuron co-stained with MAP2.

## Background

PSD-95 is a key scaffolding protein and a widely used postsynaptic marker for excitatory synapses. Within the postsynaptic density (PSD), PSD-95 assembles into dense nanodomains that recruit both AMPA and NMDA receptors in alignment with the presynaptic active zone, thereby facilitating and regulating efficient synaptic transmission. Dysregulation of PSD-95 has been associated with cognitive and learning deficits linked to neurodevelopmental disorders such as schizophrenia and autism [3].

The highly crowded environment of the PSD presents a major challenge for reliable antibody-based labeling, as the large size of full-length antibodies introduces significant steric hindrance and limits access to epitopes deep within the nanodomain. This often results in biased labeling toward peripheral molecules. Additionally, full-length antibodies introduce a linkage error of up to ~12 nm, and commonly used primary–secondary antibody complexes can extend this to ~24 nm. While negligible in diffraction-limited microscopy, such displacement becomes substantial in super-resolution techniques with sub-50 nm resolution.

To overcome these limitations, we developed Sylites using a peptide microarray approach [4]. Sylites are synthetic bidentate peptide probes with nanomolar affinity for key postsynaptic density markers: PSD-95 at excitatory synapses and Gephyrin at inhibitory synapses. Unlike conventional full-length antibodies (~150 kDa) or nanobodies (~15 kDa) [5], the ~3 kDa Sylite peptides minimize dye offset, significantly reducing linkage error and enabling precise, spot-on labeling for high-resolution microscopy. Their small size also greatly enhances tissue penetration and labeling efficiency, particularly within densely packed synaptic environments.

eSylites were designed by mimicking and dimerizing the C-terminal tail of GluN2B, a subunit of NMDA receptors. This confers nanomolar affinity and high specificity for the PDZ1 and PDZ2 domains of PSD-95 through avidity. We demonstrate that eSylites offer superior localization accuracy and structural resolution of postsynaptic nanodomains compared to conventional antibodies.

In parallel, iSylites were developed as dimers derived from the GlyR β subunit binding sequence, targeting the C-terminal E-domain of Gephyrin, which forms a dimeric structure. The modifications, together with avidity-based design, enhance binding affinity to Gephyrin up to 46,000-fold [6], enabling reliable and specific labeling of inhibitory postsynaptic densities. Together with compact synthetic probes developed by the Hamachi lab for GABA_A_Rs and GluRs [7], Sylites offer a complete toolbox for the offset-free labeling of inhibitory and excitatory neurotransmitter receptors, together with their underlying post-synaptic scaffolds.

Both eSylites and iSylites take advantage of the fractional occupancy of scaffolding protein binding sites [8,9]. While this functional selectivity is negligible in diffraction-limited imaging, it can reveal additional insights into the reserve pool of scaffold proteins when super-resolution techniques with resolution beyond 10 nm are applied. Currently, Sylites face limitations in direct stochastic optical reconstruction microscopy (dSTORM) imaging due to dense labeling, which may induce energy transfer mechanisms that compromise localization precision and photostability in dSTORM buffers [10]. Nevertheless, their fully synthetic origin and modular design make Sylites highly adaptable for specific experimental applications. In the future, advanced imaging techniques such as expansion microscopy (ExM) combined with DNA-PAINT or optimized dSTORM conditions may further enhance the utility of Sylites in nanoscale synaptic mapping.

## Materials and reagents

**Biological materials**

1. P0–P1 mouse, C57BL/6J strain

**Reagents**

1. Sylites (iSylite: Nanotag, catalog number: P4001; eSylites and a range of conjugates are also available via mail to hans.maric@uni-wuerzburg.de and jwhell@ucdavis.edu

2. Poly-D-lysine (PDL) hydrobromide (Sigma-Aldrich, catalog number: P6407-5MG, CAS number: 27964-99-4)

3. UltraPure^TM^ DNase/RNase-free distilled water (Thermo Fisher, catalog number: 10977015)

4. Gibco^TM^ HBSS, no calcium, no magnesium (Thermo Fisher, catalog number: 14170112)

5. Gibco^TM^ HBSS, calcium, magnesium (Thermo Fisher, catalog number: 24020117)

6. B-27^TM^ Plus Supplement (50×) (Thermo Fisher, catalog number: A3582801)

7. Neurobasal^TM^ Plus medium (Thermo Fisher, catalog number: A3582901)

8. GlutaMAX^TM^ supplement (Thermo Fisher, catalog number: 35050061)

9. Trypsin-EDTA (0.05%), phenol red (Thermo Fisher, catalog number: 25300054)

10. Gentamicin solution (Sigma-Aldrich, catalog number: G1397-10ML)

11. PBS, pH 7.4 (Thermo Fisher, catalog number: 10010023)

12. Pierce^TM^ 16% formaldehyde (w/v), methanol-free (Thermo Fisher, catalog number: 28906)

13. Glyoxal 40 wt. % solution in water (Sigma-Aldrich, catalog number: 128465)

14. Fish serum blocking buffer (Thermo Fisher, catalog number: 37527)

15. Triton X-100 (Sigma-Aldrich, catalog number: T9284-100ML, CAS number: 9036-19-5)

16. ProLong^TM^ Gold antifade mountant (Thermo Fisher, catalog number: P10144)

17. Paraformaldehyde, 16% w/v aqueous solution, methanol-free (Thermo Fisher, catalog number: 043368.9M)

18. Ethyl alcohol, pure (Sigma-Aldrich, catalog number: 459836-100ML)

19. Acetic acid, glacial (Sigma-Aldrich, catalog number: 695092-100ML)

20. NaOH (Fisher Scientific, catalog number: S318)

**Solutions**

1. PDL solution (see Recipes)

2. Culturing medium (see Recipes)

3. 4% paraformaldehyde (PFA) solution (see Recipes)

4. Glyoxal buffer (see Recipes)

5. Blocking and permeabilization buffer (see Recipes)

**Recipes**

**1. PDL solution**

5 mg of Poly-D-lysine hydrobromide in 50 mL of ultrapure water.

**2. Culturing medium**

**Table:** 

Reagent	Final concentration	Quantity or volume
B-27 plus supplement 50×	1×	1 mL
GlutaMAX 100×	1×	500 μL
Gentamicin	5 μg/mL	5 μL
Neurobasal Plus	n/a	48.5 mL
Total	n/a	50 mL

Prepare and equilibrate in a CO_2_ incubator one day prior to dissection.

**3. 4% PFA solution**

1 mL of 16% PFA in 3 mL of PBS, pH 7.4.

**4. Glyoxal buffer**

**Table:** 

Reagent	Final concentration	Quantity or volume
Ethyl alcohol, pure	21.6%	39.45 mL
Acetic acid glacial	0.82%	1.5 mL
Ultrapure water	n/a	141.75 mL
1 M NaOH	n/a	Titrate to pH 5
Total	n/a	~200 mL

Add 0.856 mL of 40 wt. % glyoxal solution to 10 mL of glyoxal buffer prior to fixation [11].

**5. Blocking and permeabilization buffer**

**Table:** 

Reagent	Final concentration	Quantity or volume
PBS (1×)	50%	5 mL
Fish serum	50%	5 mL
Triton X-100	0.5%	50 μL
Total	n/a	10 mL

For surface labeling with antibody, block without adding Triton X-100 and permeabilize after surface labeling.

Blocking is primarily for antibody co-staining. When using Sylites alone, serum (either fish serum or 3% BSA) can be omitted without introducing nonspecific labeling.

**Laboratory supplies**

1. Round coverslips, #1.5 18 mm (Warner Instrument, catalog number: 64-0714)

2. Microscope slides (Globe Scientific, catalog number: 1338)

3. 12-well plate (Greiner, catalog number: 665180)

4. T25 flask/50 mL conical tube (Corning, catalog number: 430639)

5. Straight forceps

6. 4” micro straight scissors, Castroviejo stainless steel (Electron Microscopy Sciences, catalog number: 72933)

8. Freer elevator/spatula

9. 60 mm Petri dish (Celltreat, catalog number: 229663)

10. Scalpel

11. Ultra-fine forceps (Excelta Corp, model: 7-SA)

12. Pipette tips

13. Parafilm

14. 150 mm Petri dish (Celltreat Scientific, catalog number: 229656)

## Equipment

1. CO_2_ incubator (Heraeus HERAcell 240 CO_2_ Incubator)

2. Dissection microscope (AmScope, model: SM-1)

3. Spectrophotometer/NanoDrop (BioTek Synergy 2 with Take3 plate)

3. Fluorescence microscope (Leica, model: TCS SP8 gated STED 3× microscope)

## Procedure

**A. Preparation of dissociated hippocampal neurons on coverslips**

*Note: All procedures involving animals followed the guidelines for the Care and Use of Laboratory Animals from the US National Institutes of Health and were approved by the Institutional Animal Care and Use committees at the University of California, Davis. If you wish to prepare the primary neuronal cultures that are part of this protocol, ensure that you have all institutional permissions from the appropriate authorities for the use of the respective laboratory animals.*

**Figure 1.**
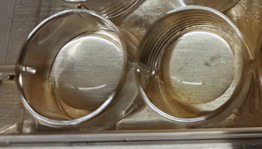
Poly-D-lysine (PDL) coating on coverslips. Limiting PDL to the coverslip surface restricts cells to the coverslip during seeding, preventing them from spreading to the bottom of the plate.

1. One day prior to P0–P1 mouse dissection, prepare coated coverslips by putting 18 mm #1.5 coverslips in individual wells of a 12-well plate.

2. Add 270 μL of PDL solution on top of the coverslips. Ensure that the solution forms a stable meniscus on top of the coverslip without spilling over to the bottom of the well, as shown in Figure 1.

3. Incubate the 12-well plate overnight at 4 °C.

4. Prepare culturing medium in a 50 mL T25 flask with a vented cap or in a 50 mL conical tube with the cap loosely closed. Incubate in a CO_2_ incubator overnight at 37 °C.

5. Before dissection, rinse the coverslips three times with ultrapure water and allow them to air-dry inside the biosafety cabinet with the lid removed. Air drying usually takes approximately 1 h and is normally completed by the time the dissection steps are finished.

6. Decapitate E18 or P0–P1 pups at the base of the skull, then stabilize the head by inserting a pair of thin straight forceps into the eye sockets (Video 1).

7. Cut the skin along the sagittal suture from the posterior to the anterior side with 3.5 mm Castroviejo micro scissors.

8. Cut the skull along the sagittal suture from the posterior to the anterior side and along the bone plate below the ears on both sides.

9. Carefully scoop out the brain by using a freer elevator or a spatula and immerse it in precooled HBSS solution without calcium and magnesium in a 60 mm Petri dish.

10. Slice off the cortex from the midbrain by using a scalpel.

**Video 1.**
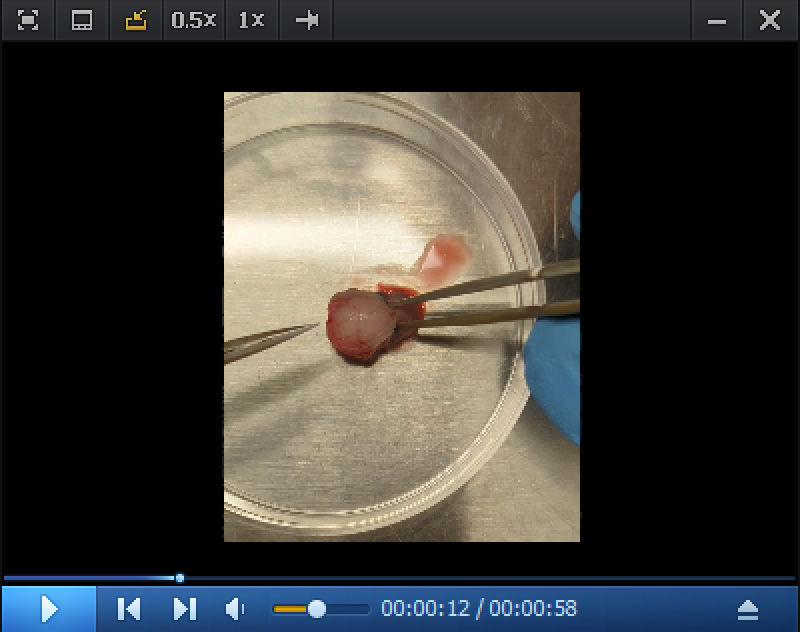
Isolation of cerebral hemispheres from P0–P1 mouse neonates

11. Using a dissection microscope, carefully peel off the meninges with ultrafine forceps (Video 2).

12. Orient the hemisphere with the dorsal side of the brain facing upward. The hippocampi should be clearly visible.

13. Remove the hippocampi from the cortex by using forceps. For detailed procedures and pictures, see [12].

**Video 2.**
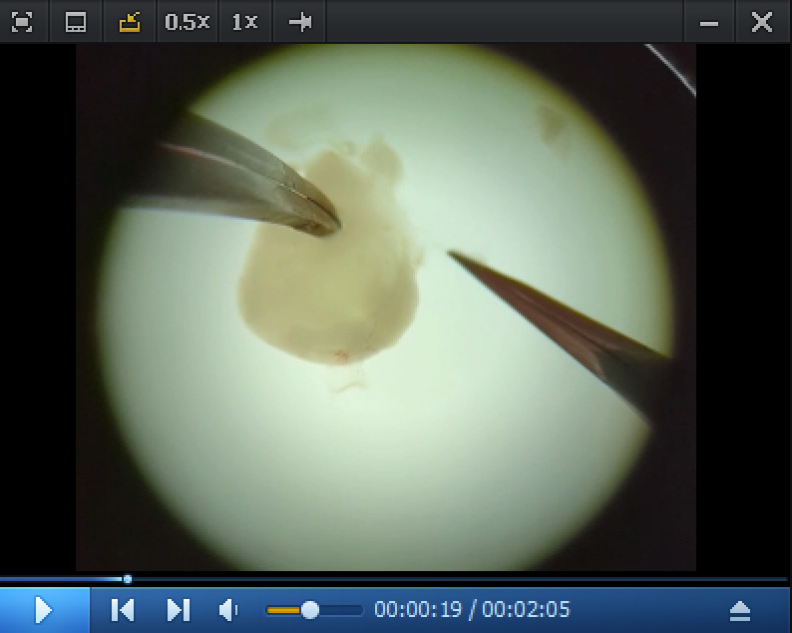
Isolation of hippocampi from cerebral hemispheres

14. Transfer the isolated hippocampi into a 5 mL conical tube containing fresh HBSS without Ca^2+^ and Mg^2+^ solution and keep on ice while dissecting the remaining animals (two pups are sufficient to prepare one 12-well plate).

15. Remove the HBSS solution and rinse with prewarmed 0.05% trypsin-EDTA solution twice.

16. Incubate the tissue in 5 mL of fresh, prewarmed 0.05% trypsin-EDTA solution at 37 °C for 15 min, gently flipping the tube midway through the incubation.

17. Remove the trypsin-EDTA solution, then quench any residual enzymatic activity by washing the tissue three times with HBSS containing calcium and magnesium.

18. Replace the solution with 1 mL of pre-equilibrated culture medium.

19. Carefully triturate the tissue using a 1 mL pipette tip trimmed to different pore sizes: 11 times with a large-pore tip, 11 times with a medium-pore tip, and 11 times with an uncut tip.

20. Allow the undissociated tissue chunks to settle by gravity for 1 min.

21. Take out the supernatant and perform a cell count using a hemocytometer with a 5–10-fold dilution.

22. Dilute cell suspension to a final density of 360,000 cells/mL with pre-equilibrated culture medium.

23. Add 250 μL of the suspended cells on top of the pre-coated coverslip. The solution should form a stable meniscus on top of the coverslip without spilling over to the bottom of the well.

24. Incubate the cells in a CO_2_ incubator for at least 2 h for the cells to adhere onto the coverslip.

25. Add 750 μL of culture medium to make up the volume to 1 mL.

26. Incubate the cell in a CO_2_ incubator for at least 14 days and refresh 40% of the culture medium every 7 days.

**B. Neuron fixation**

1. Fix the neurons at the desired days in vitro (DIV) by first removing the culture medium and rinsing twice with PBS, followed by a 15-min incubation with either 4% PFA or glyoxal solution. Ensure that cells do not dry out during solution changes. While glyoxal fixation may offer improved image quality, no significant difference in labeling efficiency has been observed between PFA and glyoxal.

2. Remove fixation solution and gently wash the cells with PBS for 5 min on an orbital shaker, repeating the wash three times.

**Pause point:** Fixed neurons can be stored at 4 °C after fixation for up to 2 weeks.

**Critical:** Sylite labeling is sensitive toward the choice of fixation reagent. eSylite labeling is sensitive to the integrity of the PDZ binding pockets. Cells stored for more than one month may exhibit collapsed binding pockets, resulting in a loss of eSylite signal, while PSD-95 antibody staining may still be detectable.

**Caution:** Glyoxal and PFA are environmentally hazardous. Quench excess aldehydes by incubating with 1 M glycine for 15 min before discarding the solution down the drain.

**C. Sylite labeling**

1. Block and permeabilize the cells using a blocking/permeabilization solution (see Recipe 5) for at least 30 min at room temperature. Cells can be kept in the blocking/permeabilization solution for 1–2 days at 4 °C.

*Note: For extracellular labeling, perform blocking and permeabilization separately, omitting Triton X-100 during the blocking step. Fish serum or 3% BSA in PBS can be used as the blocking reagent. Blocking is not necessary when using Sylites alone and is primarily required when additional antibodies are included in the labeling cocktail.*

2. Dilute the Sylite solution to a final concentration of 25–100 nM in blocking and permeabilization solution. While labeling is possible at concentrations as low as 1 nM, lower concentrations may result in incomplete labeling and reduced signal intensity. Additional antibodies can be included in the same solution for multichannel imaging.

3. Add 100μL of the Sylite-containing blocking and permeabilization solution onto a flat sheet of Parafilm placed in a 150 mm Petri dish.

4. Carefully pick up the coverslips with forceps, flip them, and place them cell-side down onto the Sylite solution for incubation. Incubate for at least 30 min at room temperature or overnight at 4°C. Incubation for 30 min or 2 h at room temperature did not show a discernible difference under diffraction-limited imaging; however, 1 h at room temperature is recommended to ensure efficient co-labeling with antibodies in the cocktail. Avoid exposing the samples to strong light after Sylite labeling. For overnight incubation, place a wet Kimwipe inside the Petri dish to maintain humidity and prevent the solution from drying out.

5. Transfer and flip the coverslip back to a 12-well plate. Gently wash the cells with PBS for 5 min on an orbital shaker; repeat the wash three times.

6. Secondary antibody labeling can be performed at this step, if needed.

**D. Imaging with fluorescence microscope**

1. For confocal/STED microscopy, add one drop of ProLong^TM^ Gold antifade mountant onto a clean microscope slide. Pick up the coverslip and gently blot the edge with a Kimwipe to remove excess PBS. Carefully place the coverslip onto the mountant, cell-side down, and avoid trapping air bubbles during mounting.

**Critical:** Sylite with sulfoCy5 labeled, designed for dSTORM, is not photostable enough even with the mountant. Imaging with the sulfoCy5 version of Sylite should be done with less laser power and multiple line average and frame accumulation to increase the signal-to-noise (S/N) ratio and signal intensity. The STAR635P version of Sylite is photostable and easier to image with confocal and STED microscopy.

2. Allow the mountant to cure at room temperature for 24 h, keeping the slide protected from light during this period.

3. Rinse the coverslip surface with ultrapure water and gently blot dry to remove any residual PBS salts, which can damage the objective lens.

**Pause point:** Mounted coverslips can be stored at 4°C in the dark for several weeks without significant signal loss.

4. Proceed with imaging according to the specific instructions for your microscope system.

## Validation of protocol

**Figure 2.**
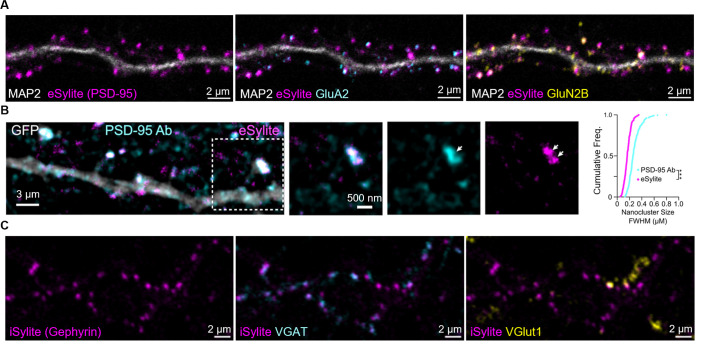
Image gallery of Sylite probes. (A) Confocal imaging of eSylite (sCy5-labeled) in 23 days in vitro (DIV) dissociated neuronal cultures, co-labeled with antibodies against MAP2, GluA2, and GluN2B. (B) tau-STED imaging of STAR635P-labeled eSylite in eGFP-transfected dissociated 21 DIV cortical neurons, co-labeled with a PSD-95 antibody visualized by Alexa Fluor 594-conjugated secondary antibody. Cumulative frequency distributions of FWHM of tau-STED-resolved nanoclusters labeled by the antibody (cyan line, n = 236 nanoclusters) and eSylite (magenta line, n = 239 nanoclusters; ***p < 0.0001, K−S test). Reprinted (adapted) with permission from [1]. (C) Confocal imaging of iSylite (sCy5-labeled) in 20 DIV dissociated neuronal cultures, co-labeled with antibodies against VGAT and VGlut1.

Images of Sylites applications in fixed primary neuron imaging are shown in Figure 2.

This protocol or parts of it has been used and validated in the following research article:

- Huhn et al. [1]. eSylites: Synthetic Probes for Visualization and Topographic Mapping of Single Excitatory Synapses. *Journal of the American Chemical Society*.
- Khayenko et al. [2]. A Versatile Synthetic Affinity Probe Reveals Inhibitory Synapse Ultrastructure and Brain Connectivity. *Angewandte Chemie International Edition*.

## General notes and troubleshooting

**General notes**

1. Cell culture conditions and seeding density significantly influence neuronal development and final image quality. As a general guideline, seed approximately 40,000 cells on 12mm coverslips, 90,000 cells on 18mm coverslips, and 200,000 cells on 25mm coverslips. Neurons older than 25 DIV may begin to degenerate, often exhibiting segmented dendrites with bubble-like structures.

2. The stock concentration of Sylite should be calibrated using a UV/Vis spectrophotometer or a NanoDrop^TM^ instrument, based on its absorption maximum. Working concentration of Sylites is optimal within 5−100 nM; low concentrations result in insufficient labeling, while high concentrations, up to 1 μM, generally have no effect but might cause higher background if washing is incomplete.

3. dSTORM imaging using sulfo-Cy5-labeled Sylite is not strongly recommended due to its extreme sensitivity to photobleaching. Significant loss of localizations can occur even during focus adjustment and TIRF angle alignment under weak laser illumination, despite the presence of Trolox, PCA, and PCD. This makes imaging conditions highly finicky and unreliable.

**Troubleshooting**

Problem 1: No staining was observed.

Possible cause: Suboptimal cell culture conditions or prolonged post-fixation storage.

Solution: Co-label with a synaptic marker antibody to confirm the health and synaptic maturity of the neurons. Perform Sylite labeling immediately after fixation to ensure the integrity of the unoccupied binding pocket. Note that hippocampal neurons are predominantly glutamatergic pyramidal neurons, while GABAergic interneurons represent a smaller population. In dissociated neuronal cultures, different cell types may localize unevenly, which can result in the absence of detectable excitatory or inhibitory synapses in certain areas. Make sure to scan over different fields in case of an inhomogeneous cell type distribution.

Problem 2: Weak and noisy image quality.

Possible cause: Suboptimal image acquisition condition.

Solution: For high noise, apply frame averaging to improve the signal-to-noise (S/N) ratio. For a weak signal, increase laser power, adjust detector gain, or enable frame accumulation to enhance image brightness without compromising resolution. If a strong reflection from the incident light is observed and cannot be eliminated by the filter cube, try applying time gating (~0.4–0.6ns gate) if your imaging system supports it. This can effectively suppress short lifetime autofluorescence and reflected excitation light, improving image contrast.
